# Comparative Analysis of the Corrosion Resistance of Titanium Alloys Intended to Come into Direct or Prolonged Contact with Live Tissues

**DOI:** 10.3390/ma12172841

**Published:** 2019-09-03

**Authors:** Florina Ionescu, Lucien Reclaru, Lavinia Cosmina Ardelean, Andreas Blatter

**Affiliations:** 1 Roctool SA, 34 Allée du Lac d’Aiguebelette, 73370 Chambéry, France; 2 Scientific Consultant Biomaterials and Medical Devices, 103 Paul-Vouga, 2074 Marin-Neuchâtel, Switzerland; 3 Department of Technology of Materials and Devices in Dental Medicine, “Victor Babes” University of Medicine and Pharmacy Timisoara, 2 Eftimie Murgu sq, 300041 Timisoara, Romania; 4 PX Group SA, 42 Blvd des Eplatures, 2300 La Chaux de Fonds, Switzerland

**Keywords:** titanium alloys, Ti6Al4V, Ti45Nb, Ti13Nb13Zr, generalized corrosion, localized corrosion, fatigue corrosion, cations release, metallic scaffolds, nanostructure

## Abstract

The evaluation of the biological safety and degradation of materials is quite important for risk assessment in various biomedical applications. In this study, two procedures were followed to characterize the corrosion resistance of different Ti-based alloys. The first one consisted of performing specific electrochemical tests (open circuit potential, linear resistance polarization, Tafel plots, potentiodynamic polarization) in order to highlight their behavior to the general and localized corrosion. The static and dynamic fatigue cycles combined with crevice corrosion conducted on a new prototype have completed the study. The second procedure followed was a cations extraction investigation (by inductively coupled plasma mass spectrometry) in order to verify the ionic permeability of the oxides layers formed on the surfaces. Optical and scanning electron microscopy were used for surface analysis. It was noticed that in these two electrolytes, the bulk Ti-based alloys presented an almost similar general corrosion behavior. The small differences of behavior for Ti6Al4V scaffolds were correlated to the surface oxidation and roughness (owing to the selective laser melting process). The Ti alloys presented no traces of localized corrosion at the end of the test. The fatigue cycles revealed that a strong and adhesive oxides film was formed during the static cycles (difficult to remove even during the depassivation steps). The concentration of cations released was at the detection limit, revealing very good passivation films, in adequacy with the all the other results.

## 1. Introduction

In terms of biomaterial applications, one of the inconvenient aspects is degradation, which occurs as a result of the material’s interaction with the human body or physiological fluids. Corrosion resistance can be considered a vital property for biomaterial components andis associated with the problem of metallic ion release, which is potentially harmful for the organism. Although Ti-based alloys exhibit good corrosion resistance owing to Ti oxides on their surface, the nature, composition, and thickness of the protective oxide depends on the environmental conditions. Degradation occurs on the biomaterial’s surfaces as a result of the chemical reactions between the oxide film and the chloride or fluoride ions in the media. Thus, the biocompatibility of materials in contact with a living tissue becomes a puzzle in the overall picture evaluating the effects of chemicals toxicity that come in contact with us [1,2,3,4].

Since 2006, Europe has had a new vision, ECHA (European Chemical Agency) [5], dedicated to the toxicology of chemicals to humans and its control, which is reinforced by the REACH regulation (registration, evaluation, authorization, and restriction of chemicals) [6].ECHA has developed a plan for the implementation of substances of very high concern (SVHC) [7], namely endocrine disruptors (ED), carcinogens, mutagens, toxics for reproduction (CMR), sensitizers, and allergens. Therefore, we have to reconsider our system for assessing the toxicology of substances, mixtures of substances, and devices in all types of environments (body, home). Among the SVHC incriminated by ECHA, the most frequent ones found in our homes are very toxic. About 4000 substances that can cause allergy or carcinogenic effects are listed. It is estimated that 15%–20% of Europe’s population is sensitized to allergens. Allergic reactions to substances in products and devices, in both professional and private life, are a significant and growing health problem affecting large parts of the European population [8].

Different chemicals from different sources, released at different times and from different places, can expose humans to a mixture of chemicals, inducing the “cocktail effect”. The way how chemicals are released from different sources, on the one hand, and how they combine to give rise to human exposure with adverse effects, on the other hand, has to be understood [9,10]. A complicating factor is that individual chemicals can become more dangerous when mixed with other chemicals, the “cocktail effect”. At the moment, there are no legal requirements for manufacturers to evaluate the combination of effects and risks of chemicals as a result of combined exposure [11,12]. However, the modalities of such an assessment are being reviewed by ECHA.

In this context, the medical devices field is confronted with observing the REACH regulations. REACH imposes the prohibition of using toxic substances such as Pb, Be, Cd, Hg, As, Se, and CrVI in medical devices and consumption goods [12,13]. Today, restriction conditions concerning the manufacture, marketing, and use of certain dangerous substances, mixtures, and articles can be found in Annex XVII REACH [14], which stipulates that these shall not be used.

The objective of our research was to perform specific tests to evaluate the corrosion behavior of three Ti-based alloys (Ti6Al4V, Ti45Nb, and Ti13Nb13Zr) in simulated artificial body fluids: plasma bone and a physiological solution (9 g/L NaCl), following a combination of two procedures according to ISO 10993-5, international standard for biological evaluation of medical devices.

The first procedure is actually a complete study involving several electrochemical methods, like open-circuit potential (OCP), Tafel plots, linear polarization resistance, potentiodynamic, and potentiostatic polarization, used to study the general and localized corrosion behavior of the Ti-based alloys. A complementary test was developed to study the fatigue corrosion static and dynamic behavior of these alloys.

The electrochemical/mechanical tests were coupled to another procedure used to identify and quantify the cations released from the materials in the solutions, using the inductively coupled plasma mass spectrometry (ICP-MS) technique. 

Complementary electrochemical tests were carried out on Ti6Al4V alloy scaffold structures from KUL Leuven and on Ti13Nb13Zr nanostructured alloy from UniVie, Vienna.

## 2. Materials and Methods

### 2.1. MaterialsTi-Based Alloys

Three types of Ti-based commercial alloys were considered in the present study: 

Ti6Al4V (ELI) [15], Ti45Nb [16], and Ti13Nb13Zr [17]. The chemical composition of the studied titanium alloys is given in Table 1. 

**Figure 1:**
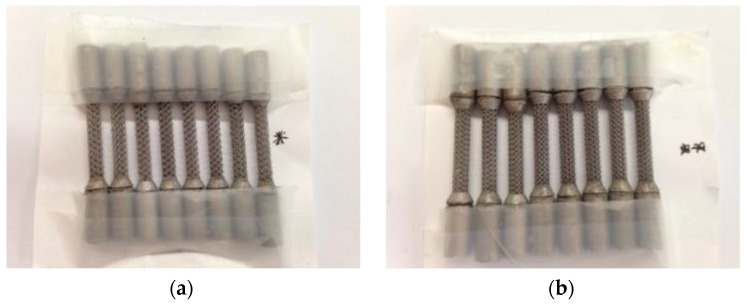
Alloy scaffolds’ structures samples: cylinders (**a**) 1000 μm and (**b**) 750 μm received from KUL Leuven, Belgium.

**Figure 2:**
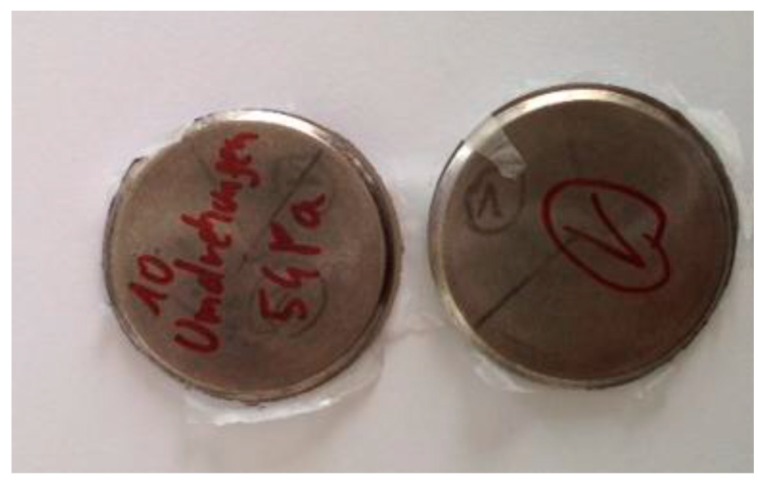
Alloy nanostructured samples: discs (1000 μm and 750 μm) received from UniVieVienna, Austria.

Complementary tests were carried out on Ti6Al4V alloy scaffold structures [18] and Ti13Nb13Zr nanostructured alloy. University KUL Leuven, Belgium provided us with Ti6Al4V alloy scaffold samples in cylindrical shapes (Figure 1). From UniVie Vienna, Austria, we received two nanostructured Ti13Nb13Zr alloy samples that were previously deformed by HPT (high pressure torsion) process, under 5 GPa and 10 rotations (Figure 2).

In order to simplify our study a codification of the Ti-based alloys samples was necessary (Table 2).

The morphology of the alloys was examined using an optical microscope (DM 4000 B/M, Leica Microsystems CMS GmbH, Wetzlar, Germany) and a scanning electron microscope (SEM JSM-6300, JEOL, Peabody, MA, USA). The samples were cut, embedded in a self-curing methyl methacrylate resin, and then polished with metallographic SiO grit-paper (600–2400). The final polishing was made on a special cloth for Ti alloys with a mixed H_2_O_2_ + SiO_2_ colloidal solution (1:2). The samples were then etched with Kroll reagent (H_2_O + HF + HNO_3_). The scaffold samples were investigated without any special preparation.

### 2.2. Procedures to Analyse the Corrosion Resistance of Ti-Based Alloys

According to ISO 10993-15 [19], in order to identify and quantify the degradation products from metals and alloys in medical devices, a combination of two procedures is recommended: 

The first procedure described is a combination of a potentiodynamic polarization test and a potentiostatic polarization test to evaluate the corrosion behavior.

The second procedure described is an immersion test to quantify the cations released.

#### 2.2.1. The First Procedure—Corrosion Resistance Evaluation

The corrosion behavior evaluation of the alloys was carried out based on specific techniques to the type of corrosion considered [20,21]:

- electrochemical evaluation of the generalized corrosion by the technique of the rotating electrode and taking into account, for evaluation, the ASTM G3-89 [22], ASTM G5-87 [23], and ASTM G59-97 [24] standards;
- pitting and crevice corrosion according to the ASTM F746-04 [25] standard;
- fatigue corrosion tests—taking into consideration that the titanium alloys considered are for orthopedic use.

**Figure 3:**
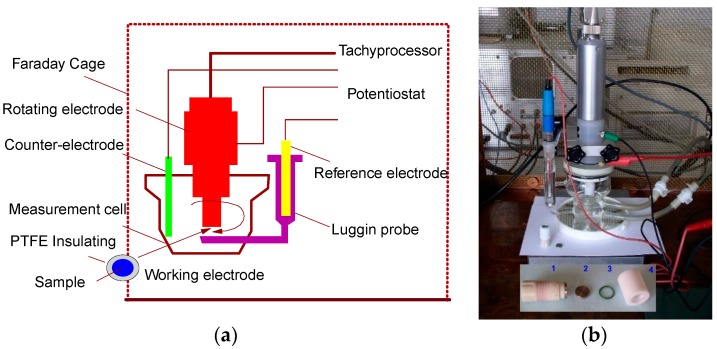
(**a**) Rotating electrode technique; (**b**) electrochemical cell used for the electrochemical tests.

The electrochemical measurements were carried out with a potentiostat model PAR 273A EG&G, Princeton Applied Research (Figure 3a), with a breakdown noise of 5 × 10^−12^ A. The electrochemical measurements were conducted in a three-electrode cell with a saturated calomel electrode (SCE) as the reference electrode and a platinum band as the counter electrode. The electrochemical cell was provided with a Luggin capillary for the reference electrode (Figure 3b). The test’s specimens (cylinders with 35 mm height and 4.5 mm diameter) served as a working electrode. To assure an accurate determination of the degradation rates, a 3.72 cm^2^ surface area was exposed to the electrolyte. The surface conditions of an alloy may affect its electrochemical behavior; consequently, in order to assess this, the samples were polished and cleaned with ethanol and acetone, and then dried with fresh air before each experiment. Afterwards, the samples were introduced into a special PTFE (polytetrafluoroethylene) port designed for rotating electrode tests.

Two types of electrolyte solutions were used for the present study:

- physiological solution—9 g/L NaCl;
- artificial plasma bone with the following composition: 6.8245 g/L NaCl, 0.2609 g/L CaCl_2_.2H_2_O, 0.4020 g/L KCl, 20.5066 g/L MgSO_4_.7H_2_O, 2.1993 g/L NaHCO_3_, 0.1674 g/L K_2_HPO_4_.3H_2_O, 0.0301 g/L NaH_2_PO_4_. H_2_O [19].

The artificial plasma bone was sterilized in order to avoid the risks of developing bacteria during the corrosion tests.

The body fluids contain a large number of cations (Na^+^, K^+^, Ca^2+^, Mg^2+^), anions (Cl^−^, HCO^3−^, PO_4_^3−^), organic acids (L-lactic, citric, D-glucuronic), and organic molecules (proteins, enzymes, lipoproteins), generally in concentration between 2 × 10^−3^ mol and 150 × 10^−3^ mol [19]. In the 1970s, several research studies stated that organic molecules did not have a significant influence on the degradation of metallic implants [26,27], but supplementary investigations highlighted that this kind of interaction, as well as the pH of the environment, should be taken into account. The two electrolytes were chosen because of their stability and their chemical composition. They do not contain any organic components that may interfere with the detection instruments [25,28]. The cell volume was 40 mL and the tests were carried out at room temperature. 

(1) General Corrosion Tests 

To evaluate the general corrosion behavior of the Ti-based alloys, several electrochemical methods were used:

- Open circuit potential (E_oc_), recorded during 15 h of immersion in the electrolyte to establish the pseudo-stationery conditions.
- Linear polarization resistance (R_p_) in the domain of Mansfeld, determined from a polarization scan between ± 20 mV vs. SCE from the E_oc_.
- Tafel plots, traced between ±150 mV/SCE close to the E_oc_ with a scan rate of 0.1 mV/s to determine the Tafel slopes (b_a_, b_c_) and the corrosion current density, i_corr_.
- Potentiodynamic polarizations plots, recorded between −1000 mV and 1000 mV/SCE with a scan rate of 0.5 mV/s to establish the breakdown potential, E_break_, and to observe the existence of a passivation domain.
- Coulometric analysis, applied to determine the quantity of the electrical charge consumed on the anodic polarization curves. The anodic domain was divided in two different subdomains: first zone: E (I = 0) to 300 mV;second zone: 300 to 600 mV.

(2) Pitting/Crevice Corrosion Tests

**Figure 4:**
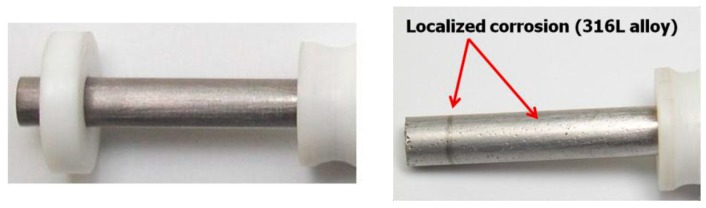
Sample used for the crevice corrosion tests.

The crevice/pitting corrosion for the Ti-based alloys was evaluated using the method presented in ASTM F746-87 standard [25]. A cylindrical sample was fitted with an inert tapered collar (PTFE) and immersed for 1 h in the electrolyte (9 g/L NaCl), in order to establish the corrosion potential (Figure 4). The crevice corrosion is then stimulated by potentiostatic polarization of the specimen to a potential much more noble than the corrosion potential (800 mV for 10 s). Immediately, the potential is decreased as rapidly as possible to one of the several preselected potentials (−300 mV, −250 mV … 750 mV) at, or more noble than, the corrosion potential. If the alloy is susceptible to pitting or crevice corrosion at the preselected potential, the polarizing current will remain at relatively high anodic values and will fluctuate or increase with time. The parameter of interest is the critical potential for crevice corrosion, E_crev_.

At the end of the test, a microscopic analysis was used to establish if the localized corrosion has occurred by pitting of the exposed surface or by preferential attack at the crevice formed by the tapered collar, or both. 

The pitting/crevice corrosion test was carried out only for the bulk samples: #1, #2, #3, and #4. Unfortunately, Ti13Nb13Zr alloy nanostructured samples could not be provided because of the sample’s dimensions. 

(3) Fatigue Corrosion Tests 

**Figure 5:**
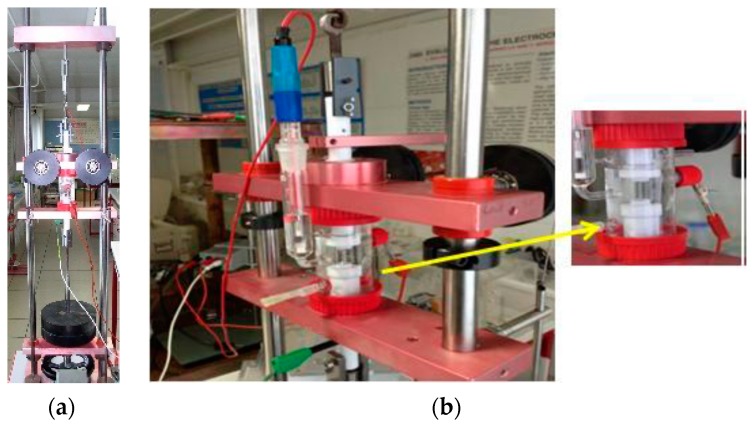
(**a**) The fatigue corrosion device; (**b**) detail of the electrochemical cell.

**Figure 6:**
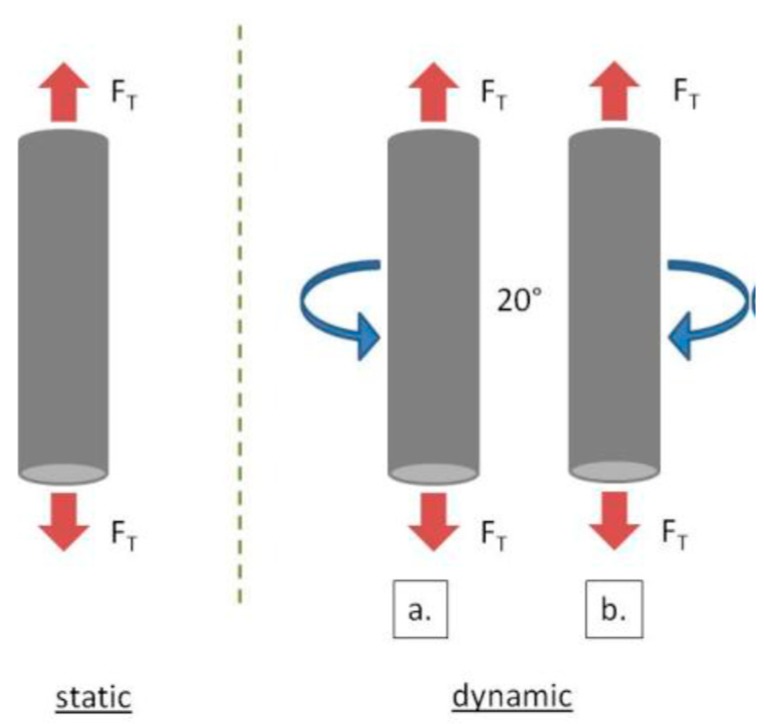
Schematic image of the two types of mechanical movements (F_T_—traction strength).

A prototype device was adapted for the fatigue corrosion tests (Figure 5). The test was carried out under static and dynamic mechanical cycles and was coupled with electrochemical cycles. For the first type, a static traction strength of 10 kg was applied and the electrochemical measurement cycles were recorded. The dynamic mechanical cycles consisted in applying the same traction strength accompanied by a 20° vertical rotation (right/left movements). The frequency of these rotations is 24 Hz and its amplitude is 1.3 cm. Figure 6 presents the schematic image of the two types of movements used for the fatigue corrosion tests.

A male female screw system is used to fix the sample into the device. The internal screw thread (female), made of copper, to ensure the electrical contact, was introduced in a Teflon holder to avoid any transfer of electrical charge. The extremities of the tested specimen were the external screws threads. Another type of protection was made using a silicone rubber coating at the interface sample/Teflon holder to avoid a possible infiltration of the electrolyte and, therefore, a galvanic couple between the copper and the tested specimen. The electrolyte was a physiological solution of NaCl at a concentration of 9 g/L in ultra-pure water, and the tests were conducted at the ambient temperature. 

The electrochemical method used was a potentiostatic one (controlled potential coulometry) that was adapted according to ASTM F746-87 [25]. This method was previously used in the laboratory to study the combined fatigue-crevice corrosion on modular hip prostheses [29]. The steps of the electrochemical cycles were also considered fit for the present study and are presented in Table 3. The choice of the electrochemical levels of these cycles is explained in a previous study [29]. Ten electrochemical cycles were performed during the mechanical cycles.

The total quantity of electrical charge consumed during the mechanical-electrochemical tests was determined by integration of the potentiostatic curves.

#### 2.2.2. The Second Procedure—Cations Release Investigations

The second procedure described, the extraction test, was used to identify and quantify the cations released from the Ti-based alloys [30].

The samples were polished and cleaned with ethanol and acetone, and then dried with fresh air. The Ti-based alloys used for the immersion test have different shapes and dimensions. The release solution volume (mL)/total sample surface (cm^2^) ratio was equal to 1. The electrolyte was the artificial plasma bone with the following composition: 6.8521 g/L NaCl, 0.2655 g/L CaCl_2_.2H_2_O, 0.404 g/L KCl, 0.2143 g/L MgSO_4_.7H_2_O, 2.1001 g/L NaHCO_3_, 0.3187 g/L K_2_HPO_4_.3H_2_O, 0.0298 g/L NaH_2_PO_4_.H_2_O [19]. The artificial plasma bone solution was sterilized by passing it through a 0.22 μm cellulose acetate membrane filter, without grid, in order to avoid the risks of developing bacteria during the corrosion testes. The specimens were placed inside an incubator at 37 °C for seven days. A blank solution was measured as a reference. 

The extraction solution was then analyzed by inductively coupled plasma atomic emission spectroscopy (ICP-AES) and inductively coupled plasma mass spectroscopy (ICP-MS). The concentrations of various metals released into solution were determined in μg/L. Given that the ratio of extraction solutionvolume/sample surface is equal to 1, the same results might be expressed in μg/cm^2^/week [31].

## 3. Results and Discussion

### 3.1. Metallographic Characterization of Ti-Based Alloys

Titanium is an allotropic element, which means it exists in more than one crystallographic form. At room temperature, titanium has a hexagonal close-packed (HCP) crystal structure, which is referred to as the α phase. This structure transforms to a body-centered cubic (BCC) crystal structure, called β phase, at 883 °C [32]. Alloying elements generally can be classified as α or β stabilizers [32,33]. Alpha stabilizers, such as aluminum and oxygen, increase the temperature at which the α phase is stable. Beta stabilizers, such as vanadium, niobium, tantalum, and molybdenum, result in stability of the β phase at lower temperatures. This transformation temperature from an α–β phase (or all α phase) to all β is known as the β_transus_ temperature. The β_transus_ is defined as the lowest equilibrium temperature at which the material is 100% β. Below the β_transus_ temperature, titanium will be a mixture of α + β, if the material contains some beta stabilizers, or it will be all α if it contains no β stabilizers [33,34].

**Figure 7:**
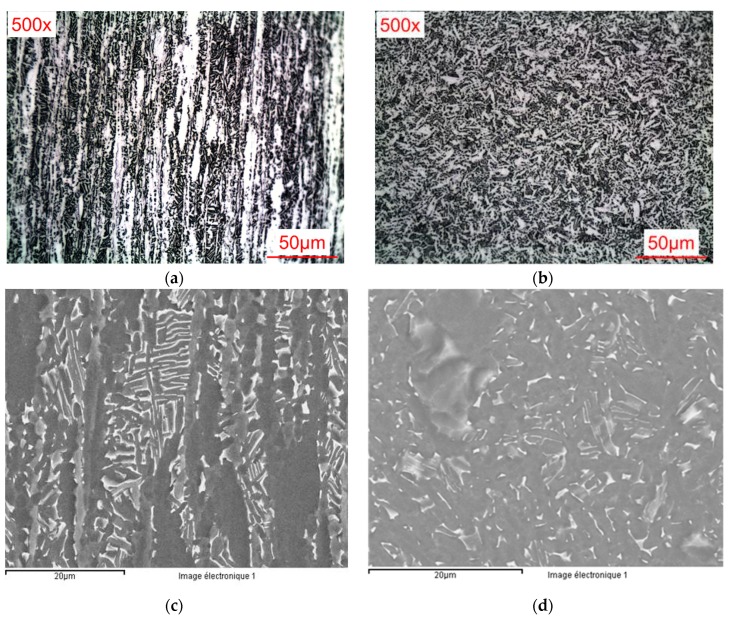
Alloy microstructure (#1): longitudinal section (**a**) optical microscopy (OM) and (**b**) scanning electron microscope (SEM) images; transversal section (**c**) OM and (**d**) SEM images.

Figure 7 shows the optical and SEM images of the Ti6Al4V alloy’s microstructure (#1), after etching. Ti6Al4V alloy has small-sized grains with clear and dark areas, which refer to the α and β phases, respectively (Figure 7b,d). The addition of alloying elements to titanium leads to a refinement of the grain sizes, thus conferring better mechanical resistance to the material. The grains were subject to deformation, and were elongated along the deformation direction (Figure 7a,c).

**Figure 8:**
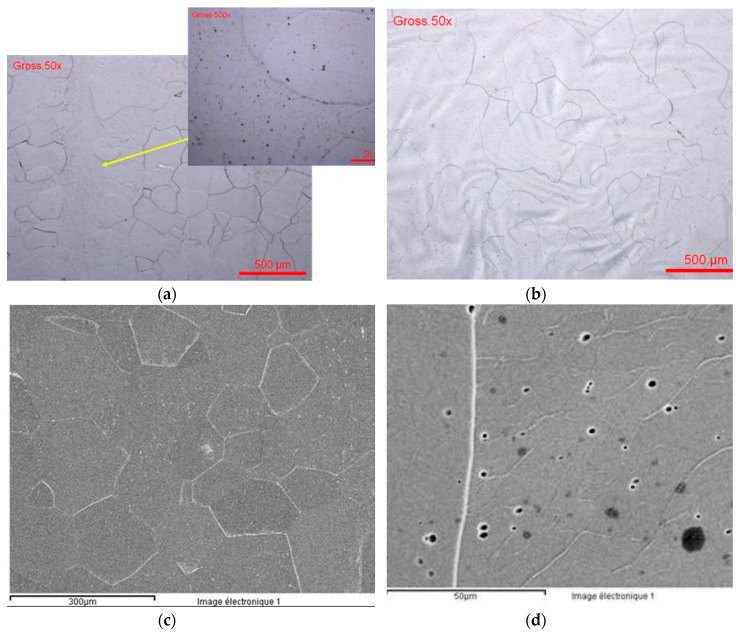
Ti45Nb alloy microstructure (#3): longitudinal section (**a**) OM and (**b**) SEM images;(#4): transversal section (**c**) OM and (**d**) SEM images.

The Ti45Nb alloy’s microstructure is presented in Figure 8. The microstructure is heterogeneous. Threadlike inclusions of niobium are observed in the niobium–titanium matrix (ripple effect or snake skin). Two different areas are noted: one with coarse polygonal grains and the other one with small grains. From the energy-dispersive X-ray spectroscopy (EDX) analysis, no difference of composition between the two areas was noted. 

Taking into consideration the results obtained in a previous study regarding the effects of crystallographic orientation of stainless steels on localized corrosion and nickel release [20], as well as the differences of Ti45Nb alloy’s microstructure, it was decided to study the corrosion behavior in two sections (longitudinal and transversal). 

**Figure 9:**
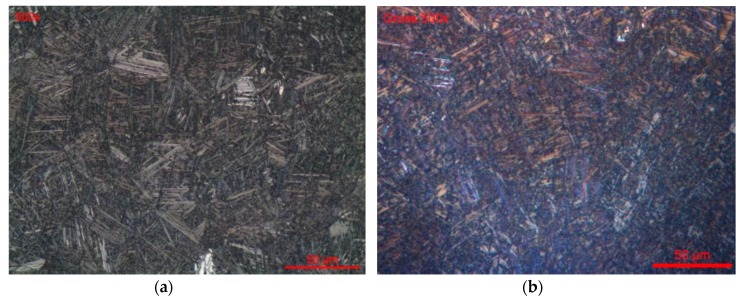

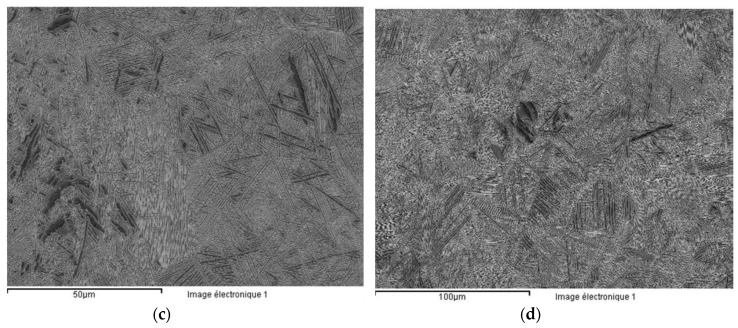
Ti13Nb13Zr alloy microstructure: longitudinal section (**a**) OM and (**b**) SEM images; transversal section (**c**) OM and (**d**) SEM images.

**Figure 10:**
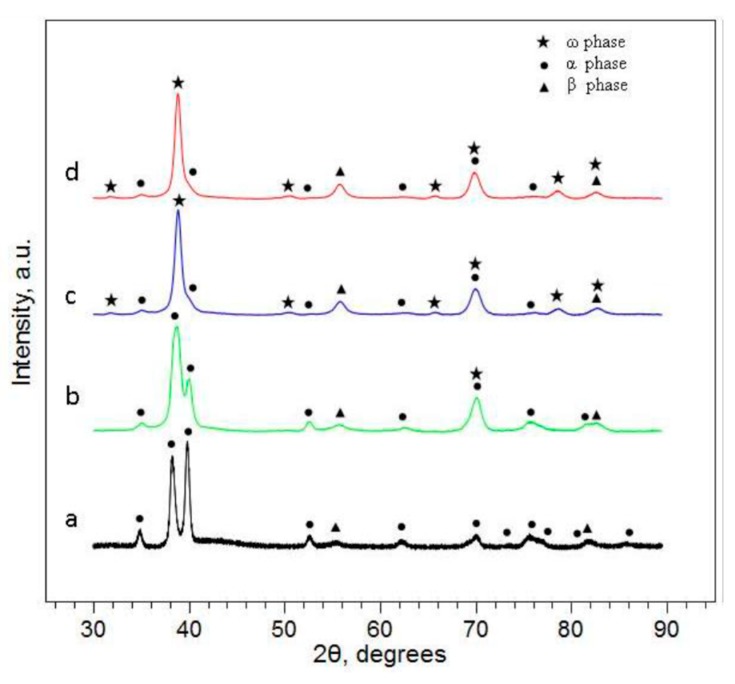
X-ray diffraction (XRD) patterns of the #2 (**a**) and #7 (**b**) 0 mm [γ = 0], (**c**) 7mm [γ = 220], and (**d**) 13 mm [γ = 408] from the center of the sample. Shear strain due to high pressure torsion (HPT) deformation was calculated using the following equation: γ = 2πrN/t, where r = distance from center to point of interest, N = number of rotations, and t = final thickness.

The bulk Ti13Nb13Zr alloy was dominated by a fine acicular α martensite in β matrix morphology (Figure 9). The presence of two phases was verified through X-ray diffraction (XRD) studies (Figure 10).

**Figure 11:**
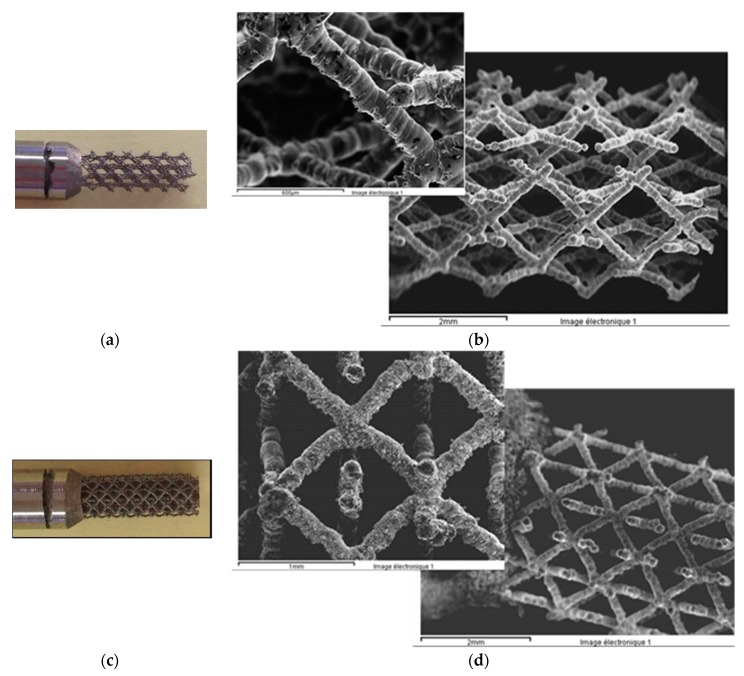
Ti6Al4V alloy scaffold structures with 1000 μm (**a**,**b**) and 750 μm (**c**,**d**) pore size.

The Ti6Al4V alloy scaffold structures were prepared by the selective laser melting (SLM) technique. This is a layer-wise material addition technique that allows generating complex 3D parts by selectively consolidating successive layers of powder material on top of each other, using thermal energy supplied by a focused and computer controlled laser beam [35,36]. The surfaces of the Ti6Al4V alloy scaffold structures were analyzed by SEM (Figure 11). The scaffolds have two different pores sizes: 1000 μm (Figure 11a,b) and 750 μm (Figure 11c,d). The strut size is 100 μm. It can be observed that the strut surface presents numerous irregularities (wholes from the removed particles). The chemical etching used at KUL Leuven on these samples does not seem to be the optimum one. According to their previous studies, a chemical and an electrochemical etching were used and the surface was smoothed with a nano-scale level roughness [37].

**Figure 12:**
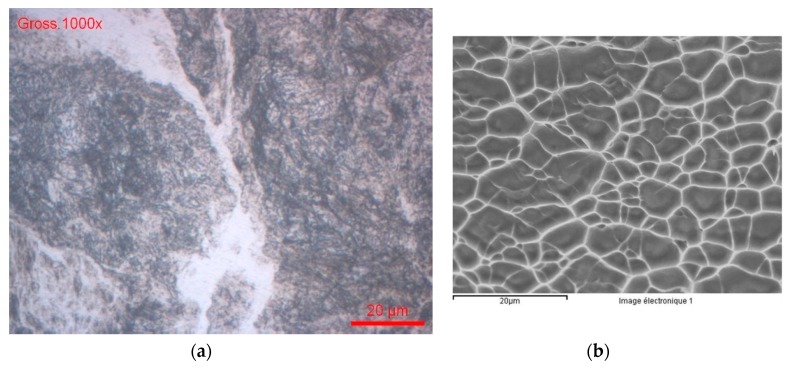
Ti13Nb13Zr nanostructured alloy (**a**) OM and (**b**) SEM images.

Ultrafine-grain sizes may be produced in bulk metals through the application of severe plastic deformation (SPD) as HPT (high pressure torsion) [38]. The Ti13Nb13Zr nanostructured alloy was prepared at the University of Vienna. Figure 12 presents the optical and electronic images of the alloy’s microstructure. During the HPT process, the α and β of the initial Ti13Nb13Zr alloys were transformed in α, β, and ω; Figure 12 (XRD analysis).

The micro-hardness of the bulk Ti-based alloys was measured (HV) and is given in Table 4. It can be noted that the longitudinal and the transversal sections have similar values of the micro-hardness. The nanostructured Ti13Nb13Zr alloy presents a higher micro-hardness value compared with the bulk alloy and similar to that of the Ti6Al4V alloy.

### 3.2. The First Procedure—Corrosion Resistance Evaluation

#### 3.2.1. Evaluation of General Corrosion Behavior of Ti-Based Alloys

(1) Evaluation of general corrosion behavior of Ti-based alloys in 9 g/L NaCl electrolyte

1) Open circuit potential (E_oc_)

**Figure 13:**
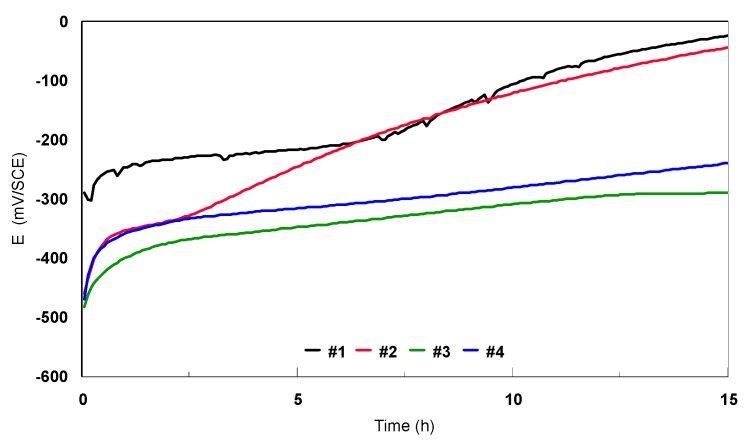
Open circuit potential vs. immersion time curves for bulk Ti-based alloys in 9 g/L NaCl electrolyte. SCE: saturated calomel electrode.

The open circuit potential is the only electrochemical technique that does not cause any disturbance to the state of the studied system. It is measured versus a reference potential, in our case, the SCE electrode. From the open circuit potential plots, it is possible to extract the first information about the electrochemical behavior of a material in contact with an aqueous corrosive environment (the preliminary transformations at the metal/electrolyte interface: corrosion, passivation). It allows to find out the required immersion time to establish the pseudo-stationery state necessary for the potentiodynamic polarization orelectrochemical spectroscopy impedance (ESI) measurements. An example of this curve, E_oc_ versus the immersion time, recorded for bulk Ti-based alloys in 9 g/L sodium chloride electrolyte, is presented in Figure 13.

The rising and steady plots (#3 alloy) show the behavior of Ti-based alloys covered with films thatthicken in time and maintain their integrity in this environment. Hoar and Fraker demonstrated that the passivation of titanium is an aging process and the latter depends strongly on the immersion period [39,40]. The oxide film formed on Ti-based alloys mainly consists of TiO_2_ and Ti_2_O_3_. The latter is a thinner and stronger film [40,41].

The open circuit potential values for the Ti-based alloys recorded after 15 h of immersion in 9 g/L sodium chloride electrolyte are presented in Table 5. In this solution, almost all the samples maintained a negative potential between −290 and −24 mV, thus situated in the anodic domain. The most negative potentials were recorded for #3, #7, and #4. 

The Ti45Nb alloy’s longitudinal and transversal sections (#3 and #4) presented a similar E_oc_. In the case of the Ti13Nb13Zr alloy, the difference between the E_oc_ of the two structures (bulk and nanostructured) was more important, about 200 mV. The E_oc_ of the nanostructured sample was more negative.

Unfortunately, this method does not permit to gain any information regarding the electrochemical kinetics or to access the corrosion rate, so complementary techniques were used.

2) Linear polarization resistance (R_p_)

The polarization resistance R_p_ can be determined, with the condition that the potential scan is between ±20 mV versus SCE from the E_oc_. The slope of the registered curve is inversely proportional to the corrosion rate. For a fixed ΔE, Δi is lower and R_p_ is higher, which means that high values of R_p_ correspond to small values of the corrosion rates.

The R_p_’s values obtained for the Ti-based alloys in 9 g/L NaCl are given in Table 5. The highest value of the R_p_ is revealed by #7 (8431 kΩ/cm^2^), followed by #1. The R_p_’s value of the Ti45Nb alloy in longitudinal section is 100 kΩ/cm^2^ higher than that calculated for the transversal section. The Ti13Nb13Zr nanostructured alloy had an extremely high R_p_ value compared with that determined for the bulk Ti13Nb13Zr alloy.

3) Tafel plots

The Tafel method can be used to determine the i_corr_, which in turn can be used to calculate the corrosion rate (if the material density and surface area are known). The curves obtained are log I versus E (potentiodynamic scan). The potentiodynamic scans are usually performed close to the open circuit potential (not more than −250 mV for a cathodic scan and 250 mV for an anodic scan). The corresponding plot must have a point where the measured current is equal to zero. The Tafel plot extrapolated to this point gives a set of co-ordinates relating to E_corr_ and i_corr_. The i_corr_ value may be calculated using the Tafel plots (b_a_ and b_c_) and R_p_ [42]. The value for b_a_ can be determined by considering the slope for the anodic portion of the curve and b_c_ for the corresponding cathodic part. The contribution of several factors like the ohmic drop, the surface activity changes (formation of a film), or the distribution of the anodic and cathodic areas can compromise the extrapolation and make it more difficult to employ.

In the present study, the pontentiodynamic scans for Tafel plots were chosen between −150 mV and 150 mV/SCE, close to the E_oc_, with a scan rate of 0.1 mV/s. The calculated values of the corrosion potential, E_corr_, and the corrosion current density, i_corr_, are given in Table 5. The corrosion potential E_corr_ characterizes the zero current electrochemical state of the Tafel scanning interface. The corrosion current density, i_corr_, characterizes the corrosion intensity at Tafel’s scanning corrosion potential (i_corr_ = i_an_ − i_cat_).

The E_corr_ is maintained in the cathodic domain; the most negative corrosion potential was recorded for sample #3, followed by #4, #7, #2, and #1. A difference of approximately 100 mV was observed between the two sections (longitudinal and transversal) of the Ti45Nb alloy. The same difference was also noted for the Ti13Nb13Zr alloys; the nanostructured alloy presented a more negative E_corr_. The corrosion current densities of the Ti-based alloys were in the same range of values, a few tens of nanoamps. For #1, the lowest value of i_corr_ (almost 3 nA/cm^2^) was determined. No significant differences were observed between the two sections of the Ti45Nb alloy or between the two Ti13Nb13Zr alloys.

4) Potentiodynamic polarization plots

The potentiodynamic polarization is probably the most commonly used electrochemical technique for measuring the corrosion resistance. It can provide significant information regarding the corrosion mechanisms and the susceptibility of specific materials to corrosion in different environments. This technique involves a variation of the working electrode potential with a selected scan rate and the resulting current is recorded as a function of potential. 

**Figure 14:**
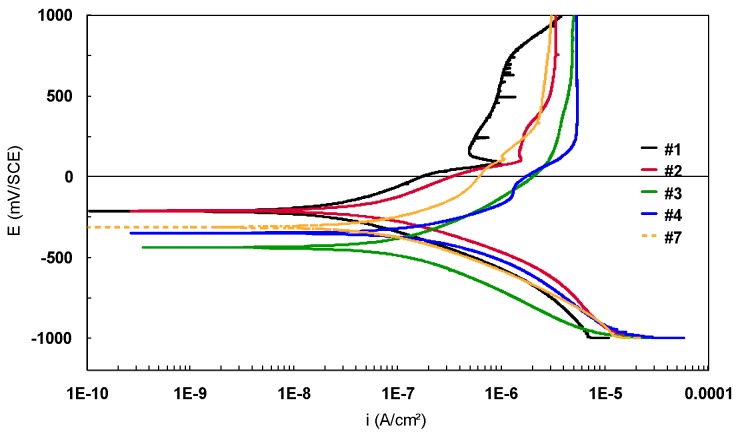
Potentiodynamic polarization curves recorded in 9 g/L NaCl electrolyte for theTi-based alloys (semi-logarithmic scale).

The potentiodynamic polarization curves in semi-logarithmic scale for Ti-based alloys (bulk materials), immersed in 9 g/L NaCl electrolyte, are presented in Figure 14. The potentiodynamic plots were recorded between −1000 mV and 1000 mV/SCE with a scan rate of 0.5 mV/s. 

According to Figure 14, a small difference between the anodic current densities of the three Ti-based alloys (a few hundreds of nanoamps) was noticed. Sample #1 is characterized by the lowest anodic current density, followed by #2 and #3. For all the alloys, an important domain of passivation (approximately 800 mV) was observed. Samples #1 and #2 presented a peak that could correspond to the degradation of a component. After that, the current densities decrease, meaning that a passive oxide layer is formed on the metallic surface. 

The two sections of the Ti45Nb alloy present similar corrosion behavior. The anodic current densities are in the same range of values, only the potential is shifted in the anodic direction for #4 compared with #3. The same observation is noted for the Ti13Nb13Zr alloys (the potential of the bulk Ti13Nb13Zr alloy is shifted in the anodic direction). 

**Figure 15:**
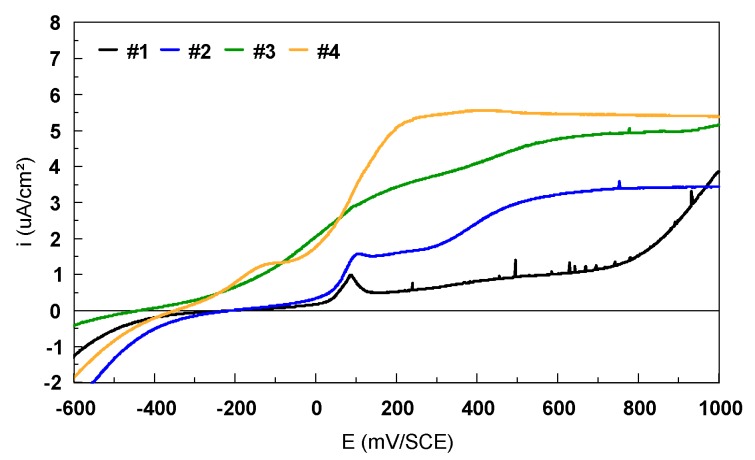
Potentiodynamic polarization curves recorded in 9 g/L NaCl electrolyte for the bulk Ti-based alloys (linear scale).

From the current–potential curves in linear scale (Figure 15), the breakdown potential, E_break_, can be also identified. The breakdown potential, E_break_, is the potential for which the anodic current increases strongly. The range of the potential situated between the corrosion potential (E_corr_ or E_(i=0)_) and E_break_ represents the immunity zone in which the corrosion is weak, even insignificant. The more this zone is extended, the less the alloy is likely to be found in a situation where, by polarization or by galvanic effect resulting from the presence of another alloy, it can be led to being strongly corroded. In the case of Ti-based alloys, it was very difficult to determinate the E_break_ for all the samples.

5) Coulometric analysis

The coulometric analysis consists of integration of the current quantity consumed during the anodic polarization. The anodic domain was divided in two different subdomains: first zone: E_(I=0)_ and 300 mV; second zone: 300 and 600 mV.

After integration, the consumed electrical charge is given in mili Coulombs (Table 5). The coulometric analysis for the two zones confirms the previous results: the best behavior is revealed by #1, #7, and #2. The consumed electrical charges were more important in the second zone than in the first one. An insignificant difference between the electrical charges consumed by the two Ti13Nb13Zr alloys during the polarization was noted. A more important quantity of electrical charge was calculated for the transversal section of the Ti45Nb alloy than for the longitudinal section. 

(2) Evaluation of general corrosion behavior of Ti-based alloys in artificial plasma bone 

1) Open circuit potential (E_oc_)

**Figure 16:**
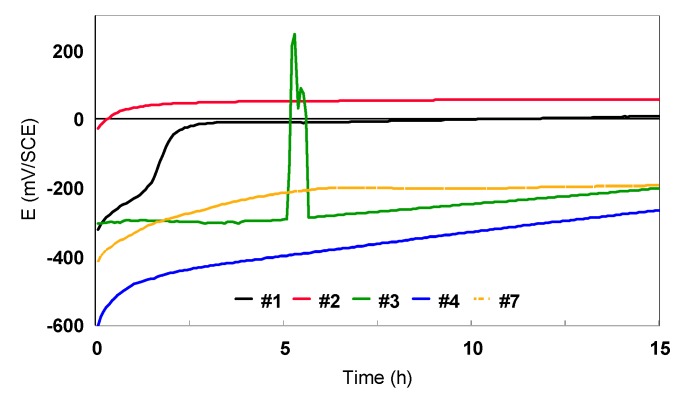
Open circuit potential vs. immersion time curves for bulk Ti-based alloys in artificial plasma bone.

The curves for all the Ti-based alloys in artificial plasma bone are presented in Figure 16. The values of the E_oc_ recorded after 15h of immersion in artificial plasma bone are presented in Table 6. Same as in the sodium chloride solution, almost all the samples maintained a stable and negative E_oc_. The #1 and #2 samples recorded positive potential values. 

Samples #3 and #4 (Ti45Nb alloy longitudinal and transversal sections) presented similar E_oc_values (−200 and −266 mV/SCE, respectively). The Ti13Nb13Zr alloy (#2) had a positive potential value, four times higher than the one recorded for the nanostructured one, and thus is situated in the cathodic domain.

2) Linear polarization resistance (R_p_)

The R_p_ values recorded for the Ti-based alloys in artificial plasma bone are presented in Table 6. Sample #7 had the highest R_p_ value, followed by samples #3 and #4. The lowest R_p_ value was revealed for #1. For the Ti45Nb alloy in the longitudinal section, a R_p_ value twice higher than for the transversal section was determined. As in the case of 9 g/L NaCl electrolyte, the R_p_ value of the nanostructured Ti13Nb13Zr (#7) was very high compared with the one determined for the bulk alloy (#2). 

3) Tafel plots

The following information can be extracted from Table 6: sample #1 has the most electropositive corrosion potential; all the other samples have the corrosion potential in the range of −235 to 145 mV/SCE. Sample #2 has the most negative E_corr_. The two sections of the Ti45Nb alloy have similar values of E_corr_. The current densities are in the same range of values (few tens of nA/cm^2^), only #1 has a higher i_corr_ value (218 nA/cm^2^).

4) Potentiodynamic polarization plots

**Figure 17:**
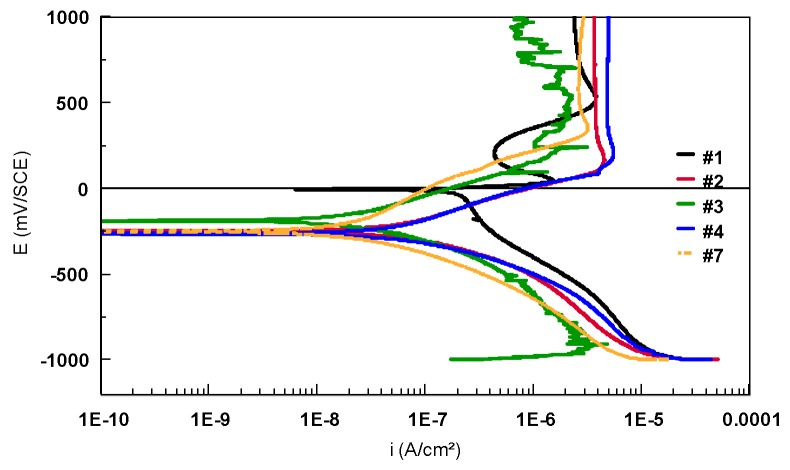
Potentiodynamic polarization curves recorded in artificial plasma bone for bulk Ti-based alloys (semi-logarithmic scale).

The potentiodynamic polarization curves in semi-logarithmic scale for Ti-based alloys (bulk) in artificial plasma bone are presented in Figure 17. As in the case of the first type of electrolyte, the potentiodynamic plots were recorded between −1000 mV and 1000 mV/SCE with a scan rate of 0.5 mV/s. The curves shapes are very different from one alloy to another. Sample #1 (Ti6Al4V alloy) presented a similar curve with the one recorded in 9 g/L NaCl electrolyte, with a peak that could correspond to a degradation component, followed by a passivation domain. Sample #2 hasrecorded the largest passivation domain, but the anodic corrosion densities are a little bit more important than those of the other alloys. Strong variations of anodic current densities were observed in the plot recorded for #3 (Ti45Nb longitudinal section), associated with pitting corrosion. The fluctuations of the cathodic current densities are related to the cathodic reaction—hydrogen evolution—during the test.

The transversal section of the Ti45Nb alloy does not present the same corrosion behavior as the longitudinal section;on the contrary, the recorded plot presents a very large passivation plateau (Figure 17).

A small difference was observed between the two curves recorded for the Ti13Nb13Zr alloys. The nanostructured samples presented lower anodic current densities values than the bulk alloy. This could be associated to the microstructure modifications generated by the HPT process, namely the morphology and the appearance of the new ω phase.

5) Coulometric analysis

As in the case of the first electrolyte, the consumed electrical charge is lower in the first zone than in the second zone, except for #3 (546.8 mC/mm^2^ in zone I vs. 1.165 mC/mm^2^ in zone II), which means that the surface’s attack, pitting corrosion, had probably occurred during zone I. 

The Ti6Al4V alloy scaffold structures have a diamond-like unit cell with a strut size of 100 µm and pore size of 750 or 1000 µm. As in the case of bulk materials, the corrosion behavior of these structures was evaluated in two electrolytes: 9 g/L NaCl and artificial plasma bone. All the electrochemical parameters are presented in Table 7.

Regarding the two scaffold structures in 9 g/L NaCl, a few observations are to be made. It was noted that the potential increased in the first period of time and remains constant afterwards. The value recorded after 15 h of immersion for #5 was lower and remained in the anodic domain, compared with #6, whichpassed in the cathodic domain. The difference between the two values was almost 100 mV. From the Tafel plots, E_corr_ and i_corr_ were calculated. A difference of a few hundreds of nanoamps was noted for the corrosion current density of the two samples (338.9 nA/cm^2^ for #5 and 21.93 nA/cm^2^ for #6). This difference was also observed for R_p_ values, #6 had a R_p_ value four times higher than #5. The electrical charge consumed during the second zone was more important than during the first one. 

In artificial plasma bone electrolyte, the values of the E_oc_ recorded after 15 h of immersion are similar (−120 mV for #5, −136 mV/SCE for #6, respectively). For both structures, the values of the corrosion potential and the corrosion current density determined from the Tafel plots are also in the same range. The R_p_ determined for #6 (750 µm pores size) is twice as low as the R_p_ of #5 (1000 µm pore size). Regarding the consumed electrical charge, the two scaffold structures presented the same tendency: more important values are calculated for the second zone than for the first zone. Sample #6, which has a larger surface, consumed more electrical charge than sample #5. 

This behavior is related to the metallic surface preparation after the SLM process. It was noted that, after etching, the Ti6Al4V alloy particles were not totally removed from the sample’s surface. 

**Figure 18:**
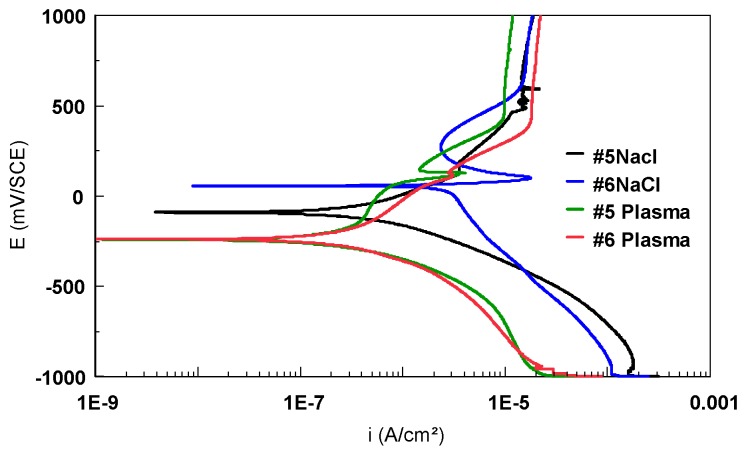
Potentiodynamic polarization curves recorded for the Ti6Al4V scaffold samples in 9 g/L NaCl and artificial plasma bone (semi-logarithmic scale).

The curves presented a peak (more or less important) that could correspond to a component degradation (Figure 18). Afterwards, the anodic current densities increased, forming an important passivation domain (~500 mV).

(3) Evaluation of crevice/pitting corrosion behavior of Ti-based alloys

This method is applied only to passive metals and alloys, and is used to rank surgical implant alloys in order of their resistance to localized corrosion [25]. The parameter of interest, the critical potential for crevice corrosion, E_crev_, is defined as the highest (most noble) preselected potential at which crevice surfaces repassivate after the stimulation step (at 800 mV for 10 s). 

Concerning this ASTM standard, it should be noted that the method was designed to reach conditions that are sufficiently severe to cause breakdown of at least one alloy (316L), currently acceptable for surgical implant use. It should also be noted that these alloys, which suffer pitting or crevice corrosion during the more severe portion of the test, do not necessarily suffer localized corrosion when placed within the human body as a surgical implant [25].

**Figure 19:**
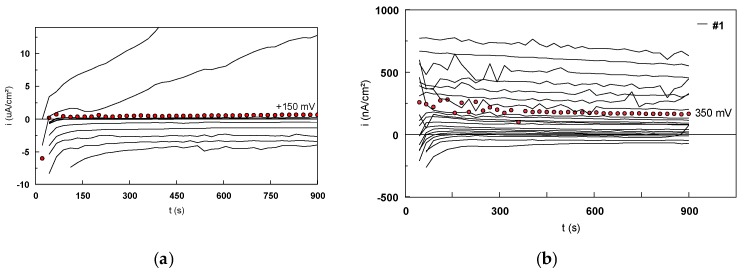

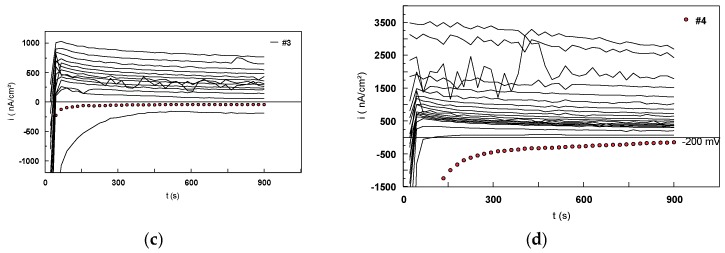
Polarization current densities recorded for different alloys, during 15 min, at preselected potentialsand above E_oc_, in a solution of 9 g/L NaCl: (**a**) 316L alloy [43] and Ti-based alloys: (**b**) #1, (**c**) #3 and (**d**) #4.

In the case of the 316L alloy (Figure 19, the E_crev_ can be easily determined. At the preselected potential of 200 mV, it can be observed that the current densities are very high, reaching typical values for localized corrosion, the surface is covered with pits and the collar removal also reveals traces of crevice corrosion. The highest preselected potential at which the pitted surface repassivates after the stimulation step is 150 mV (for 316L). 

In Figure 19, we remark that small variations of the current densities, passing from the cathodic into the anodic domain, are recorded at the preselected potentials. The E_crev_ for the Ti-based alloys is superior to 750 mV. Above the red marked potential, a possible ’’attack’’ of the metallic surface should be taken into consideration. At the same time, it should not be forgotten that the Ti-based alloys are repassivable materials and the TiO_2_ oxides quickly form at their surface [39,40].

A significant difference was observed between the two Ti45Nb alloy sections. The current densities values are in the order of µA/cm^2^ for the transversal section compared with those recorded for the longitudinal section, nA/cm^2^ (Figure 19c,d).

This difference could be caused by a direct relation with the microstructure of the transversal and longitudinal sections, or it could remain within the margins of experimental errors. Reclaru and al. showed, their study regarding the behavior of stainless steels specimens in aggressive environments (Fujayama–Meyer and Carter–Brugirand solutions), that the cross-sectional cuts have a substantially higher sensitivity to corrosion phenomena compared with longitudinal cuts [20].

The Ti-based alloys surfaces were analyzed after the crevice/pitting corrosion tests and no traces of localized (pitting or crevice) corrosion were observed, meaning that, even at these high values, the Ti45Nb (transversal section) alloy is able to repassivate. 

**Figure 20:**
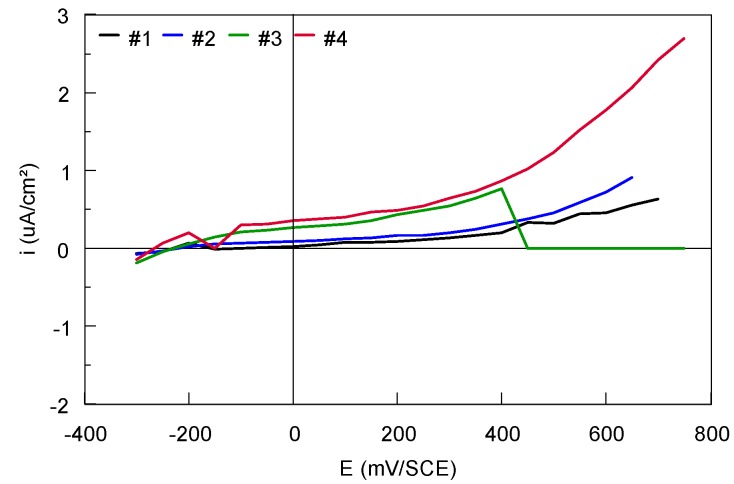
Potentiostatic polarization plots, current value recorded after 15 min, versus the preselected potentials for bulk Ti-based alloys.

The new “polarization curves” are obtained if the final value of the current recorded at the end of each plot of the potentiostatic curve is plotted as a function of the preselected potential (Figure 20) [17]. These new plots are specific for the localized corrosion behavior of Ti-based alloys.

#### 3.2.2. Evaluation of Fatigue Corrosion Behavior of Bulk Ti-Based Alloys

**Figure 21:**
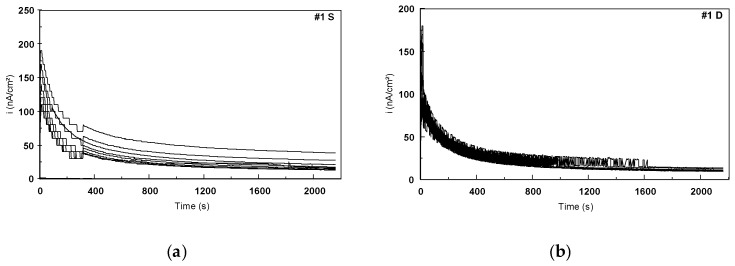
Potentiostatic curves recorded for the level of 600 mV/SCE for #1 in 9 g/L NaCl corresponding to (**a**) static and (**b**) dynamic mechanical cycles.

Two types of tests, static and dynamic mechanical cycles, were performed in 9 g/L NaCl electrolyte to evaluate the fatiguecorrosion behavior of the Ti-based alloys. These tests were coupled with the electrochemical measurements cycles (Table 3). The potentiostatic plots were recorded at each potentiostatic level: 600, 650, 700, and 750 mV. The depassivation step at −700 mV potentiostatic level was chosen to evaluate the repassivation capacity of the Ti-based alloys. Figure 21 shows the potentiostatic curves recorded at 600 mV level for #1 (Ti6Al4V alloy) during the ten electrochemical cycles for both the static and dynamic mechanical cycles. 

It should be noted that the quantities of current densities recorded during the potentiostatic scan were in the order of nano-amps. The quantities of current recorded during the dynamic mechanical cycles were inferior to those recorded during the static ones. 

The electrical charge consumed for the fatigue corrosion tests was determined from the integration of each potentiostatic plot. 

**Figure 22:**
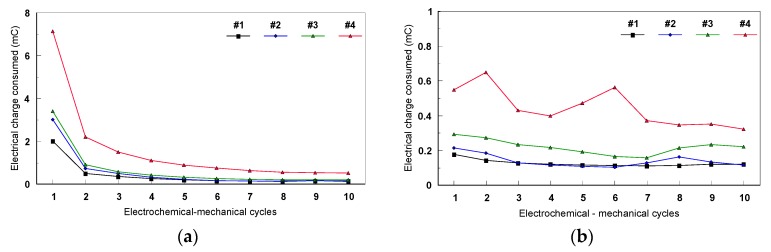
The fatigue corrosion behavior of Ti-based alloys in 9g/L NaCl during the mechanical cycles: (**a**) static and (**b**) dynamic cycles.

**Figure 23:**
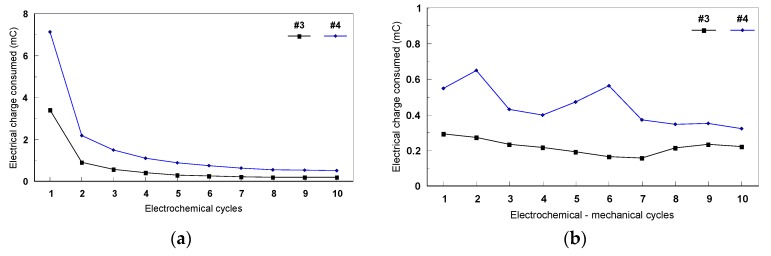
The fatiguecorrosion behavior of Ti45Nb alloy (longitudinal and transversal sections) in 9 g/L NaCl during the mechanical cycles: (**a**) static and (**b**) dynamic.

All the values of consumed electrical charge (mC) were determined for both types of mechanical cycles. Figure 22 presents the evolution of the consumed electrical charge during the two types of mechanical cycles (static and dynamic). In the case of the static cycles, a decrease of the electrical charge during the first four cycles was observed for all the Ti-based alloys, then it remains almost constant while increasing the mechanical cycle’s number. The values of the electrical charge recorded during the static mechanical tests for #4 (Ti45Nb alloy in transversal section) were more important than those of the others samples (Figure 23).

During the dynamic cycles, the values of the consumed electrical charge were between 150 and 700 mC, much smaller than those determined for the static tests. For each tested specimen, the dynamic test followed the static one. Therefore, their specific evolution of the consumed electrical charge and the difference between their values could be explained by the fact that during the static cycles, a passive film (generally the TiO_2_-anatase) is being formed and reformed, after the depassivation step at −700 mV, on the metallic surfaces. Also, when the dynamic mechanical cycles were performed, the surfaces were already covered with a thin and adherent oxide film.

**Figure 24:**
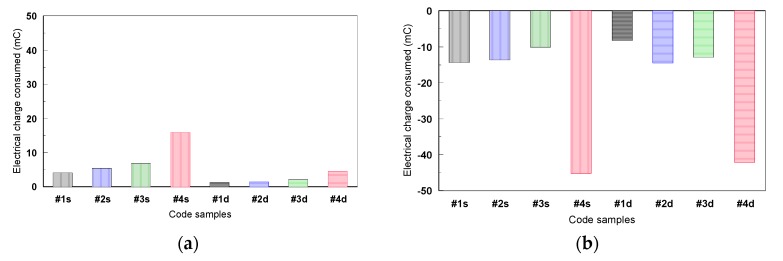
The total electrical charge consumed for the fatigue corrosion tests in 9 g/L NaCl during (**a**) the passivation and (**b**) the depassivation steps (s: static and d: dynamic mechanical cycles).

Figure 24 shows the results obtained in the mechanical-electrochemical tests during the passivation steps (potentiostatic levels: 600, 650, 700, and 750 mV) and the depassivation step at −750 mV. It was noted that the total electrical charge consumed during the passivation steps was smaller compared with the one consumed during the depassivation step, meaning that the TiO_2_ film formed on Ti alloy’s surface during the passivation steps is strongly adherent, and that it is necessary to double the electrical charge to remove it.

Analyzing the sum of electrical charges consumed during the entire test period, it was observed that the #1 (Ti6Al4V alloy) presents the best corrosion behavior and #4 (Ti45Nb alloy transversal section) presents the worst corrosion behavior.

**Figure 25:**
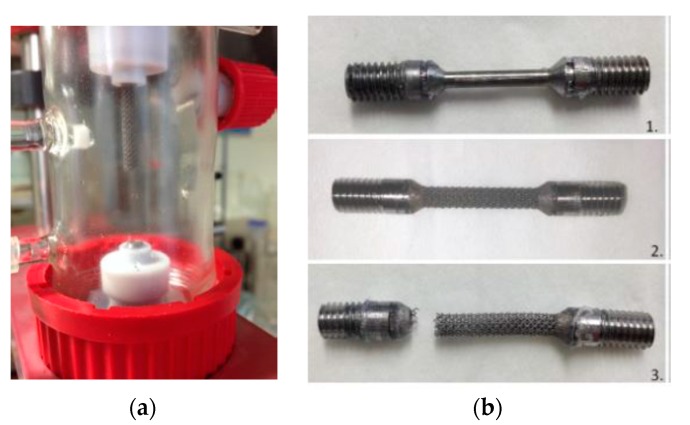
(**a**) Failure of the Ti6Al4V alloy scaffold specimen before fatigue corrosion tests; (**b**) Ti-based alloys samples before and after test.

The Ti6Al4V alloy scaffold structures were machined and prepared for the fatigue corrosion tests in the same way as the bulk alloys. Unfortunately, the fatigue corrosion tests were not conducted on these samples because they broke before starting the tests (Figure 25a). The failure was localized at the interface bulk /scaffold structure (Figure 25b). 

**Figure 26:**
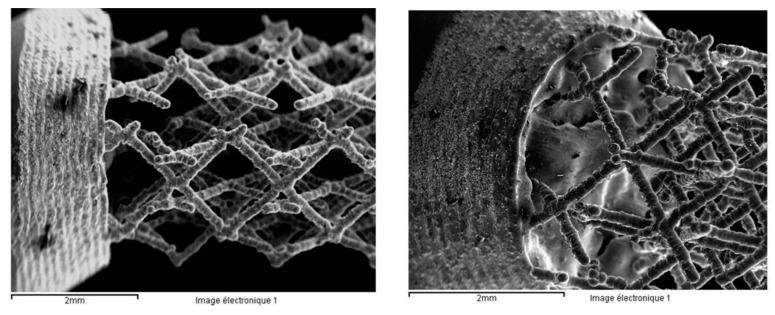
SEM images of the Ti6Al4V alloy scaffold structure taken before any machining or cleaning process.

Two factors could contribute to the failure of the samples before the fatigue corrosion tests:

- The sample was not well melted on the bulk parts during the SLM process because of the specific design. Figure 26 presents the SEM images of the scaffold structure before testing.
- The structure was also weakened during etching, the machining process, and cleaning in the ultra sound bath.

### 3.3. The Second Procedure—Cations Extraction

Various mechanisms, like corrosion, wear, stress corrosion, and fatigue corrosion, affect the metals released from orthopedic implants. The quantity of the released metal changes markedly depending on the nature and strength of the metal–oxide bond, structure (vacancies, interstitial elements, degree of ordering), role of alloying element, composition, and thickness of oxide films [40,44]. 

In Table 8, the average values of the chemical elements concentrations released from the Ti-based alloys studied are presented. No important differences were observed for the released elements between the Ti-based alloys. Almost all the values of the identified released cations were at the limit of detection (Al, Cr, Cu, Fe, Ni, Ti, V, As, Be, Cd, Hg, Li, Mo, Nb, Pb, Sb).

Sample #3 (Ti45Nb transversal section) released an important quantity of Ba (27 µg/L), Co (0.85 µg/L), and Zn (510 µg/L). Zn was also released in high quantities from the other samples.

The fact that almost all values of the released cations are at the detection limit could have a direct relation with the solution aggressiveness. According to EN 71-3:2013, the extraction solution used for these tests is hydrochloric acid 0.07 N.

**Figure 27:**
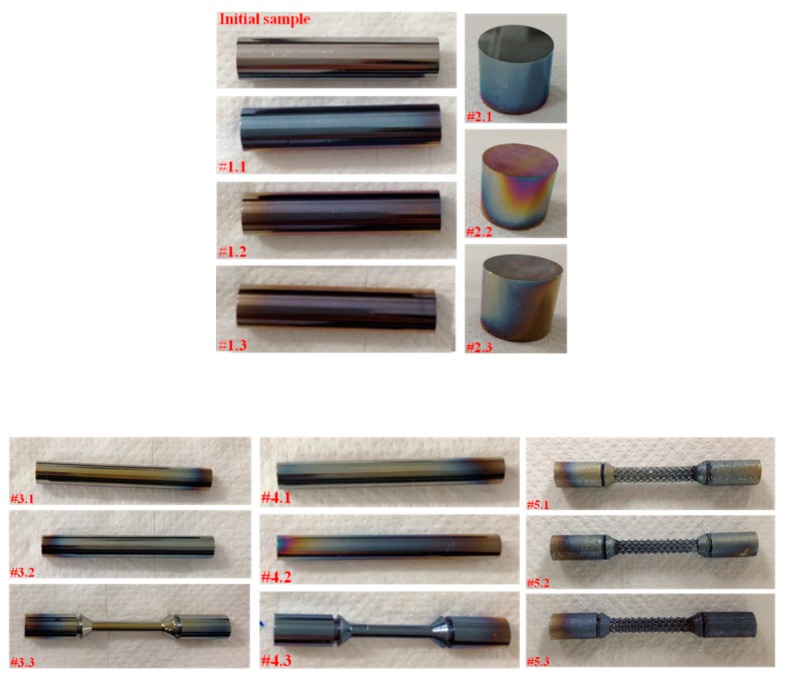
Titanium alloys after the extraction test.

After seven days of immersion at 37 °C, a coloration, which resembles tarnish films, was observed on the specimen’s surface (Figure 27). The tarnish is defined as a surface discoloration or a slight loss of the surface, generally of chemical origin [45]. In the case of dental alloys, in order to evaluate their tarnish resistance, the specimens are immersed in physiological solutions (isotonic sodium chloride solution, Ringer’s solution) or artificial saliva solutions for long cycles [46]. Takemoto et al. [47] studied the tarnish of Ti alloys immersed in alkaline and peroxide containing denture cleanser. They noted that Ti6Al4V and Ti6Al7Nb alloys showed a marked discoloration compared with pure titanium, which is the consequence of the oxidation reaction due to peroxide. Song et al., in their tarnish study, induced by Actinobacillus actinomycetem comitans serotype b on titanium and its alloys, observed that the surface color was changed to yellow-green-red ranges after two weeks of immersion [48]. For Ti, the tarnish film is a thin oxide one and its color is influenced by the alloying elements and the surface contaminants. 

A more detailed analysis of an oxide film formed on each metal surface seems to be necessary to verify its chemical composition and to validate the hypothesis of tarnish films.

## 4. Conclusions 

Two different procedures were used to study the behavior of Ti-based alloys (Ti6Al4V, Ti45Nb, Ti13Nb13Zr) in a physiological solution of 9 g/L NaCl and in artificial plasma bone. The first procedure was a combination of electrochemical techniques to study the corrosion (general and crevice/pitting) behavior of Ti-based alloys coupled with static and dynamic mechanical cycles to evaluate their fatigue corrosion resistance.

In the two environments, the bulk Ti-based alloys presented almost similar general corrosion behavior. After 15 h of immersion, a pseudo-stationary state was reached and the polarization measurements could be performed. The corrosion potentials of the Ti-based alloys remained in the cathodic domain (exception #1 in the artificial plasma bone). The corrosion current densities were found to be in the same range of values (nanoamps/cm^2^). The R_p_ values are different from one media to another; but in both cases, the Ti13Nb13Zr nanostructured alloy had the highest value, followed by Ti13Nb13Zr (bulk) and Ti6Al4V alloys. The electrical charge consumed varies between 0.3 to 3 mC. The two Ti45Nb alloy sections presented a similar behavior, except the longitudinal section in artificial plasma bone, which showed a crevice corrosion behavior. This behavior could be related to the surface state (the surface was polished with 600 grind paper).

Small behavior differences were noted for the two Ti6Al4V scaffold structures, when different electrolytes were used, which were correlated with the SLM process, with the oxidation state of the surface and its roughness. 

The crevice corrosion potential was determined to be higher than 750 mV (the last preselected potential), but it should be taken into consideration that a “possible attack” of the metallic surface could have happened at smaller potentials. A significant difference of the current densities was noted between the two Ti45Nb alloy sections. In this case, the current densities recorded for Ti45Nb in transversal section were higher compared with the longitudinal section. In the end of these aggressive tests, no traces of localized corrosion (crevice and /or pitting) were noted. 

The fatigue corrosion behavior of the Ti-alloys during the two types of mechanical cycles was different. During the static cycles, the quantity of consumed electrical charge decreases with the increase of the mechanical cycle numbers and remains constant in time compared with the dynamic cycles, in which case it is lower and almost constant from the beginning to the end of the test. The metallic surface has already been covered with a strong and adherent Ti oxides film. 

The total quantity of the consumed electrical charge during the depassivation step was two to three time higher compared with the one determined for the passivation steps, meaning that the Ti oxides films were difficult to remove from the surface, which reveals good electrochemical resistance. 

Unfortunately, the Ti6Al4V scaffold structures failed before the fatigue corrosion tests. To perform these type of tests, another structure design must be taken in consideration for the Ti6Al4V alloy scaffold. 

The second procedure consisted of an immersion test used to identify and quantify the released cations. Several elements were identified in the artificial plasma bone solution: Al, As, Ba, Be, Cd, Co, Cr, Cu, Fe, Hg, Li, Mo, Nb, Ni, Pb, Sb, Sn, Sr, Ti, V, and Zn. Almost all the released cation concentrations (µg/L) were at the detection limit, which reveals very good passivation films and subsequent corrosion resistance, in accordance with the results obtained in the first procedure.

## Figures and Tables

**Table 1:** Composition in % weight of the alloys tested.

Alloy	C	N	O	H	Fe	Al	V	Nb	Zr	Ti
Ti6Al4V	0.0110	0.0060	0.1100	0.0034	0.1200	6.0800	3.9300	-	-	rest
Ti45Nb	0.0200	0.0200	0.1600	0.0050	0.2500	-	-	45.5200	-	rest
Ti13Nb13Zr	0.0500	0.0150	0.1100	0.0010	0.0550	-	-	13.7300	13.7300	rest

**Table 2:** Samples’ codification. HPT: high pressure torsion.

Code	Alloy	Sample Characteristics
#1	Ti6Al4V	bulk, longitudinal section (L)
#2	Ti13Nb13Zr	bulk, longitudinal section (L)
#3	Ti45Nb	bulk, longitudinal section (L)
#4	Ti45Nb	bulk, transversal section (T)
#5	Ti6Al4V	scaffold structure with 1000 μm pores size
#6	Ti6Al4V	scaffold structure with750 μm pores size
#7	Ti13Nb13Zr	nanostructured by HPT process

**Table 3:** Composition of an electrochemical measurement cycle [29].

No.	Step	Potential (mV)	Time (min)
1.	Excitation	800	1
2.	Potentiostatic level	600	36
3.	Excitation	800	1
4.	Potentiostatic level	650	36
5.	Excitation	800	1
6.	Potentiostatic level	700	36
7.	Excitation	800	1
8.	Potentiostatic level	750	36
9.	Potentiostatic level	−700	20

**Table 4:** Ti-based alloys’ micro-hardness values, (HV).

Alloy	Hardness (HV)
#1	337 ± 4
#2	258 ± 8
#3	187 ± 4
#4	189 ± 3
#7	362 ± 11

**Table 5:** Electrochemical parameters determined for Ti-based alloys in 9 g/L NaCl.

Sample	E_oc_ mV	E_corr_ mV	i_corr_ nA/cm^2^	b_a_ mV/dec	b_c_ mV/dec	R_p_ kΩ.cm^2^	Coulometric Analysis	
							Zone I mC/cm^2^	Zone II mC/cm^2^
#1	–24	–10.75	2.83	49.34	150.50	1105.7	0.354	0.518
#2	–44	–17.60	13.22	77.59	134.10	359.8	0.801	1.585
#3	–290	–305.5	26.62	153.80	196.40	247.8	1.854	2.568
#4	–240	–232.2	29.66	146.50	171.50	254.6	2.416	3.308
#7	–259	–124.3	14.02	134.30	151.80	8431.5	0.728	1.477

**Table 6:** Electrochemical parameters determined for Ti-based alloys in artificial plasma bone.

Sample	E_oc_ mV	E_corr_ mV	i_corr_ nA/cm^2^	ba mV/dec	bc mV/dec	Rp kΩcm^2^	Coulometric Analysis	
							Zone I mC/cm^2^	Zone II mC/cm^2^
#1	10	8.9	218.2	54.53	303.80	100.3	0.418	1.542
#2	58	−235.9	34.14	163.20	166.30	271.5	2.189	2.320
#3	−200	−145.6	12.61	113.70	190.90	652.3	546.8	1.165
#4	−266	−205.5	19.35	1.970	212.80	332.5	2.554	3.005
#7	−193	−180.0	12.29	337.40	180.70	1563	0.458	1.741

**Table 7:** Electrochemical parameters determined for Ti6Al4V scaffold samples in 9 g/L NaCl and in artificial plasma bone.

Sample	E_oc_ mV	E_corr_ mV	i_corr_ nA/cm^2^	ba mV/dec	bc mV/dec	R_p_ kΩcm^2^	Coulometric Analysis	
							Zone I mC/cm^2^	Zone II mC/cm^2^
*in 9 g/L NaCl*								
#5	−86	−96.53	338.9	314.10	163.50	125.0	2.027	7.108
#6	38	3.258	21.93	154.20	109.40	670.1	2.360	4.039
*in artificial plasma bone*								
#5	−120	−138.7	49.61	265.60	113.90	442.2	1.154	5.204
#6	−136	−117.2	106.4	174.50	117.50	187.9	2.364	10.24

**Table 8:** Quantities of cations released from Ti-based alloys (µg/L).

Cation	Blanc	#1	#2	#3	#4	#5	#6	#7
As	<10	<10	<10	<10	<10	<10	<10	<10
Ba	2.4	2.2	2.0	27.2	2.1	1.66	2.0	1.63
Be	<0.1	<0.1	<0.1	<0.1	<0.1	<0.1	<0.1	<0.1
Cd	<0.2	<0.2	<0.2	<0.2	<0.2	< 0.2	<0.2	<0.2
Co	0.3	<0.2	<0.2	0.85	<0.2	<0.2	<0.2	<0.2
Hg	<0.5	<0.5	<0.5	<0.5	<0.5	<0.5	<0.5	<0.5
Li	<1	<1	<1	<1	<1	<1	<1	<1
Mo	<0.2	<0.2	<0.2	<0.2	<0.2	<0.2	<0.2	<0.2
Nb	<0.02	<0.02	<0.02	<0.02	<0.02	<0.02	<0.02	<0.02
Pb	<0.2	<0.2	<0.2	<0.2	<0.2	<0.2	<0.2	<0.2
Sb	<0.2	<0.2	<0.2	<0.2	<0.2	<0.2	<0.2	<0.2
Sn	<0.2	< 0.2	<0.2	<0.2	<0.2	<0.2	<0.2	<0.2
Sr	12.0	21.6	18.6	16.6	17.3	15.0	20.30	16.0
Zn	11.0	116.60	120.0	510.0	120.0	110.0	116.60	120.0
Al	8.20	<20	<20	<20	<20	<20	<20	<20
Cr	0.9	<2	<2	<2	<2	<2	<2	<2
Cu	<4	<8	<8	<8	<8	<8	<8	<8
Fe	<4	<8	<8	<8	<8	<8	<8	<8
Ni	<4	<8	<8	<8	<8	<8	<8	<8
Ti	<2	<2	<2	<2	<2	<2	<2	<2
V	<0.8	<0.8	<0.8	<0.8	<0.8	<0.8	<0.8	<0.8
Zr	<4	<8	<8	<8	<8	<8	<8	<8
